# Competing endogenous RNAs regulatory crosstalk networks: The messages from the RNA world to signaling pathways directing cancer stem cell development

**DOI:** 10.1016/j.heliyon.2024.e35208

**Published:** 2024-07-26

**Authors:** Hamid Aria, Mahdieh Azizi, Shima Nazem, Behnam Mansoori, Farzaneh Darbeheshti, Anoosha Niazmand, Abdolreza Daraei, Yaser Mansoori

**Affiliations:** a Noncommunicable Diseases Research Center, Fasa University of Medical Sciences, Fasa, Iran; b Department of Immunology, School of Medicine, Isfahan University of Medical Sciences, Isfahan, Iran; c Department of Clinical Biochemistry, Faculty of Medicine, Tehran University of Medical Sciences, Tehran, Iran; d Pediatrics Department, School of Medicine, Fasa University of Medical Sciences, Fasa, Iran; e Department of Radiation Oncology, Dana-Farber Cancer Institute and Brigham and Women's Hospital, Harvard Medical School, Boston, MA, USA; f Department of Genetics and Molecular Biology, School of Medicine, Isfahan University of Medical Sciences, Isfahan, Iran; g Cellular and Molecular Biology Research Center, Health Research Institute, Babol University of Medical Sciences, Babol, Iran; h Department of Medical Genetics, School of Medicine, Babol University of Medical Sciences, Babol, Iran; i Department of Medical Genetics, Fasa University of Medical Sciences, Fasa, Iran

**Keywords:** Cancer stem cells, Competing endogenous RNAs, ceRNA, lncRNA, Micro-RNA

## Abstract

Cancer stem cells (CSCs) are one of the cell types that account for cancer heterogeneity. The cancer cells arrest in G0 and generate non-CSC progeny through self-renewal and pluripotency, resulting in tumor recurrence, metastasis, and resistance to chemotherapy. They can stimulate tumor relapse and re-grow a metastatic tumor. So, CSCs is a promising target for eradicating tumors, and developing an anti-CSCs therapy has been considered. In recent years competing endogenous RNA (ceRNA) has emerged as a significant class of post-transcriptional regulators that affect gene expression via competition for microRNA (miRNA) binding. Furthermore, aberrant ceRNA expression is associated with tumor progression. Recent findings show that ceRNA network can cause tumor progression through the effect on CSCs. To overcome therapeutic resistance due to CSCs, we need to improve our current understanding of the mechanisms by which ceRNAs are implicated in CSC-related relapse. Thus, this review was designed to discuss the role of ceRNAs in CSCs' function. Targeting ceRNAs may open the path for new cancer therapeutic targets and can be used in clinical research.

## Highlights

• Cancer stem cells (CSCs) can generate non-CSC progeny, resulting in tumor recurrence and chemo-resistance.
• One of the mechanisms by which CSCs cause tumor relapse is competing endogenous RNA (ceRNA).
• ceRNA affects gene expression via competition for microRNA (miRNA) binding.
• Aberrant ceRNA expression is associated with tumor progression.
• Targeting ceRNAs may open the path for new cancer therapeutic targets.

## 1 Background

Despite substantial achievements in cancer research, there is still a lack of understanding of the basic mechanisms of cancer development, demanding further research. While cancer begins with a single mutant cell, it is highly heterogeneous and includes a wide range of proliferative and differentiated cells. Tumor progression, metastasis, therapeutic resistance, and recurrence are thought to be caused by this heterogeneity [1]. Among the various cell types that account for cancer heterogeneity, the presence of cancer stem cells (CSCs) is crucial [2]. CSCs are a small number of tumorigenic cells inside the tumor mass that remain dominant during rapid tumor cell proliferation. They arrest in the G0 phase and generate non-CSC progeny by self-renewing and pluripotency activity, resulting in tumor recurrence, metastasis, and chemoresistance [[3], [4], [5]].

CSCs possess some of the same markers (CD133 and CD44) as normal stem cells or signaling pathways involved in self-renewal and differentiate into different cell types [[6], [7], [8]]. But CSCs are around 10-fold more tumorigenic than non-CSC populations, for example, in pancreas cancers [9]. CSCs frequently exhibit the re-expression of embryonic markers such as octamer-binding transcription factor 4 (OCT4), SRY [sex-determining region Y]-box 2 (SOX2), Nanog, and Dnmt1. In comparison to CD44‾/CD133‾ cells, the Notch pathway was active in prostate CSCs. In addition, prostate CSCs expressed stem cell markers such as SOX2, CMYC, OCT4, krüppel-like factor 4 (KLF4), CD90, and stage-specific embryonic antigen-1 (SSEA-1) [10]. CSCs have metabolic profiles different from those of terminally differentiated tumor cells and are found in specialized hypoxic microenvironments that contribute to long-term maintenance [[11], [12], [13]]. A key component of the transcriptional program that keeps stem cells in a self-renewal undifferentiated pluripotent state is the high-mobility-group DNA-binding protein SOX2. LIN28 is the core RNA-binding pluripotency stem cell factor that overexpressed and promotes cancer growth and metastasis. It is implicated in breast CSC maintenance and collaborates with KLF4, SOX2, and NANOG to promote pluripotency [[14], [15], [16], [17], [18]]. Krüppel-like factor 4 (KLF4) is a transcription factor with zinc fingers conserved throughout evolution. It controls a variety of biological functions, including cell growth, proliferation, and differentiation [19], and acts as an oncogene highly expressed in more than 70 % of breast tumors [20]. OCT4 is a transcription factor expressed in various cancers and maintains the proliferation and pluripotency (stemness) of CSCs [21].

A multifunctional cell membrane receptor known as CD44 has been identified as a cancer-causing or stem cell marker [22]. CD44 regulates a wide range of physiological processes, including cell adhesion, lymphocyte activation, cell migration, cell proliferation, angiogenesis, and tumor metastasis [23]. In the liver, gastric, breast cancer, and acute myeloid leukemia (AML), CD44 is a CSC marker [24]. Also, prostate CSCs have a CD44^+^/α2β1^high^/CD133^+^ phenotype and constitute about 0.1 percent of prostate cancer cells. In comparison to CD44^‾^/α2β1^low^/CD133^‾^ cells, CSCs have a greater capacity for proliferation and invasion [25,26]. CD44 modulates the expression of CDC42, a Rho GTPase involved in cell motility and cell-cycle progression, by binding and suppressing miR-216a, miR-330, and miR-608 [27].

Zinc finger E-Box binding homeobox 1 (ZEB1) and ZEB2 have been identified as the principal regulators for maintaining stem cell characteristics. By suppressing promoter activity, transcription factors ZEB1 and ZEB2 can start an EMT process by reducing E-cadherin transcription [28].

## 2 CSCs, what is their importance?

CSCs are thought to be responsible for tumor growth with a low rate of proliferation, making them resistant to radiotherapy and chemotherapy [29]. CSCs are less resistant to anti-cancer therapies than conventional cancer cells [30]. Conventional therapies are effective in the early stages of cancer treatment, but they fail to target and eradicate CSCs, which contribute to chemoresistance and tumor recurrence [31]. CSCs frequently express ATP-binding cassette (ABC) transporters, which are multidrug resistance proteins (MDRs) that export drugs from cancer cells and promote drug resistance [32]. Chemotherapy and radiotherapy cause DNA damage and apoptosis, but CSCs, by increasing DNA repair capacities, can effectively prevent cancer cells from apoptosis [33]. So, CSC is a promising target for eradicating malignant tumors; hence developing an anti-CSC method has become a top priority in cancer treatment.

CSCs are a scarce cell population within a tumor, but as the tumor progresses, this fraction can increase to over 30 %, and this increase is associated with therapy resistance [[34], [35], [36], [37], [38]]. Dysregulation of self-renewal is thought to be the first stage of tumorigenesis [39]. Furthermore, CSCs have a critical role in epithelial-mesenchymal transition (EMT) and are believed to be the primary sources of tumorigenesis, development, metastasis, and relapse [25,[40], [41], [42], [43]]. Because CSCs have a high self-renewal potential, they can stimulate tumor relapse at the tumor site and re-grow a metastatic tumor at a distant location [35,[44], [45], [46]]. As a result, tumors with a higher CSC marker have a weaker prognosis than tumors with a lower CSC population [47].

## 3 Non-coding RNAs and CSCs

The molecular mechanisms driving the development of CSC properties are currently unknown, but new research suggests that microRNAs (miRNAs) may play a role in CSC regulation [48]. miRNAs are a type of non-coding RNA that are 19–25 nucleotides RNA [49]. They regulate gene expression through base pairing with its mRNA, inhibit translation, and increase mRNA degradation [50,51]. miRNAs are involved in a variety of physiological and pathological processes, including tumorigenesis, metastasis, and therapy resistance [[52], [53], [54], [55]]. Tumor stage, metastasis, relapse, therapeutic resistance, and survival have all been associated with miRNA expression profiles [56,57]. miRNAs have been associated with the modulation of CSC features such as cell-cycle progression, differentiation, migration, invasion, and EMT [58]. As a result, studying miRNAs that impact drug sensitivity could be a valuable tool for better understanding the mechanisms behind drug resistance and cancer therapy.

**Fig. 1:**
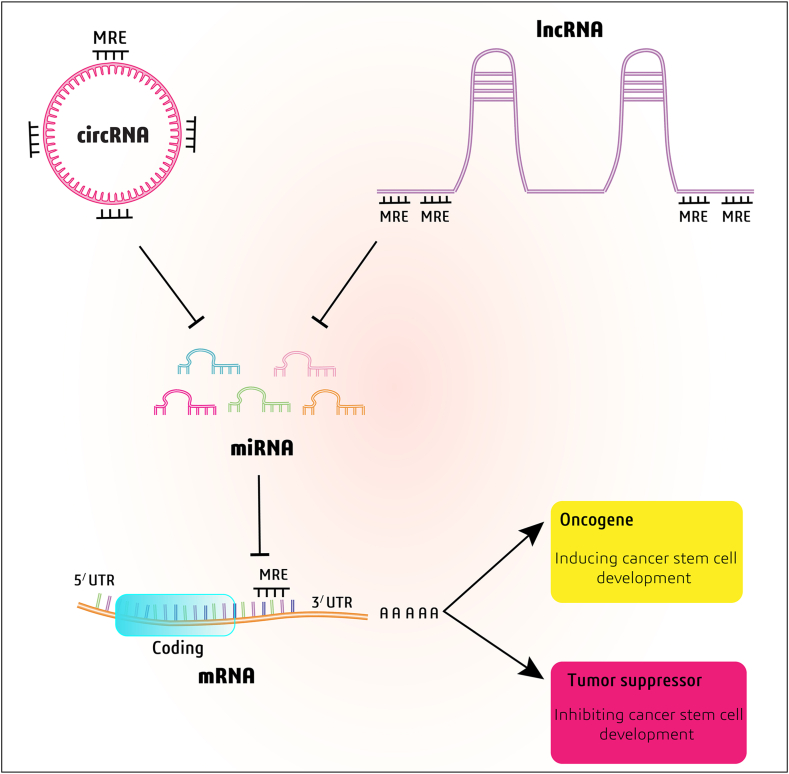
ceRNA's mechanism of action. lncRNA and circRNA may have shared miRNA binding sites known as miRNA response elements (MREs) and in this way can sponge miRNAs competitively and regulate each other or downstream target genes indirectly.

RNA molecules with shared microRNA response elements (MREs) can regulate each other by competing for binding to microRNA. This process is termed competing endogenous RNA (ceRNA) regulation [59] (Fig. 1). This theory suggests that there are interaction networks among all types of RNA transcripts through competing for identical sequences in miRNAs to modulate each other's expression [60,61]. According to the proposed ceRNA theory, RNAs may interact with each other and influence biological processes independent of protein translation [62]. In recent years ceRNAs have emerged as an important class of post-transcriptional regulators that affect gene expression via competition for miRNAs binding [63]. Extensive studies have found that aberrant ceRNA expression is associated with cancer progression, prognosis, and pathogenesis by modulating the expression of critical tumorigenic and tumor-suppressive genes [64,65].

Long non-coding RNAs (lncRNAs) are widely transcribed RNA molecules with more than 200 nucleotides but do not code for proteins [66]. LncRNAs play a role in a variety of biological processes, including epigenetic regulation of gene expression, stem cell pluripotency regulation, and so on [[67], [68], [69]]. Long intervening/intergenic non-coding (LINC) RNAs, intronic ncRNAs, and sense or antisense lncRNAs are all types of lncRNAs with various genomic locations regarding genes and exons [70]. Through competitive miRNA binding, lncRNAs act as the key regulatory mechanisms affecting many targeted genes.

Circular RNA (circRNA) is a special type of non-coding RNA with no free end produced from pre-mRNAs [71]. They have a role as regulators of gene expression and miRNA inhibition. So, they are involved in many disease pathogenesis. Recent studies have shown that circRNAs may be involved in EMT and tumorigenesis [71]. It has been demonstrated that circRNAs have several conserved miRNA response elements (MREs), compete with mRNAs as miRNA sponges, and upregulate the target gene's expression to control gene expression [72].

**Fig. 2:**
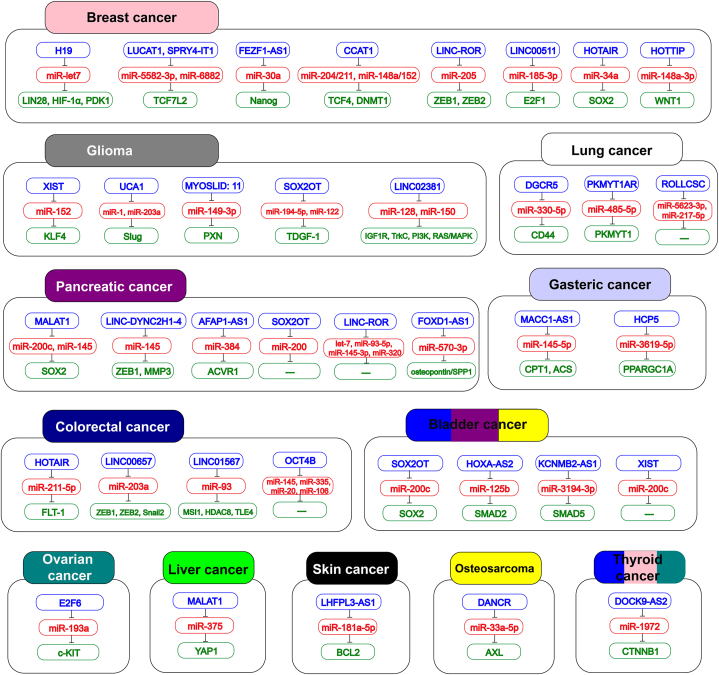
A list of lncRNAs that promote CSCs and their miRNA and mRNA targets in various types of cancer.

**Fig. 3:**
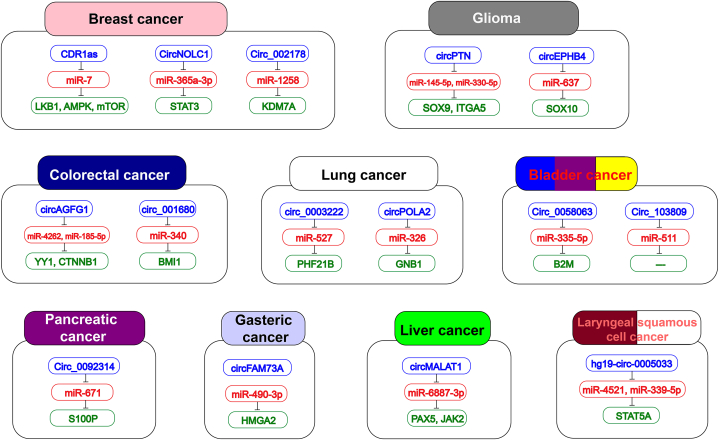
A list of circRNAs that promote CSCs and their miRNA and mRNA targets in various types of cancer.

**Fig. 4:**
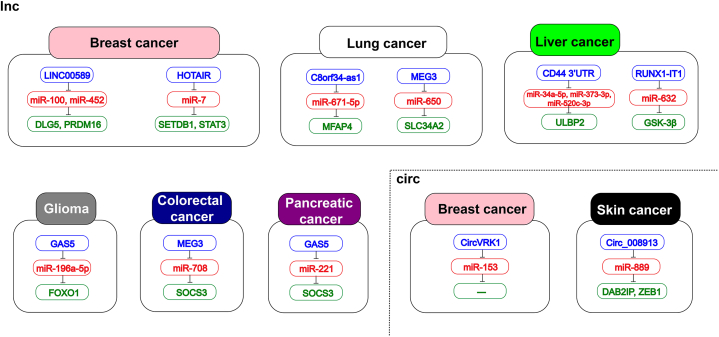
A list of lncRNAs and circRNAs that inhibits CSCs and their miRNA and mRNA targets in various types of cancer.

To overcome therapeutic resistance, we need to improve our current understanding of the mechanisms by which ceRNAs are implicated in CSC-related relapse. Thus, this review was designed in order to discuss the role of ceRNAs in CSCs function (Table 1). Also, list of CSCs-promoting lnc-RNAs, CSCs-promoting circ-RNAs, and CSCs-inhibiting lnc-RNAs/circ-RNAs along with their targets, are shown in Fig. 2, Fig. 3, Fig. 4 respectively.

**Table 1:** ceRNA regulation network related to cancer stem cell (CSC) properties.

Cancer	ncRNA	miRNA	mRNA	Pathway	CSC marker	CSC properties	Promote/inhibit CSC features	Ref.
Breast	H19	miR-let7	LIN28	–	LIN28, OCT4, SOX2, Nanog, ALDH1, POU5F1	Stemness markers, sphere formation, migration	Promote	[73]
	H19	miR-let7	HIF-1α, PDK1	Glycolysis	CD44, ALDH1, c-Myc, POU5F1, LIN28, OCT4	Stemness markers, sphere formation	Promote	[74]
	LUCAT1	miR-5582–3p	TCF7L2	Wnt/β-catenin	CD44, OCT4, SOX2, Nanog, ABCC1, Wnt1, HIF-1α	Stemness markers, sphere formation, chemoresistance	Promote	[75]
	SPRY4-IT1	miR-6882	TCF7L2	Wnt/β-catenin	CD44, OCT4, c‐Myc, Nanog, SOX2, ABCC1, ABCB1	Stemness markers, sphere formation, chemoresistance	Promote	[76]
	FEZF1-AS1	miR-30a	Nanog	–	CD44, Nanog, OCT4, SOX2	Stemness markers, sphere formation, migration, invasion	Promote	[77]
	CCAT1	miR-204/211, miR-148a/152	TCF4, DNMT1	WNT/β-catenin	CD44, Nanog, OCT4, SOX2, ALDH1	Stemness markers, sphere formation, migration, invasion	Promote	[78]
	LINC-ROR	miR-205	ZEB1, ZEB2	–	CD44, ZEB1, ZEB2, Fibronectin, N-cadherin, Vimentin, α-SMA	Sphere formation, EMT, migration, invasion, metastasis	Promote	[79]
	LINC00511	miR-185–3p	E2F1	–	OCT4, Nanog, SOX2	Stemness markers, sphere formation, invasion	Promote	[80]
	LINC00589	miR-100, miR-452	DLG5, PRDM16	–	CD44, CD133, Nanog, OCT4, SOX2	Stemness markers, sphere formation, chemoresistance	Inhibit	[81]
	STARD13-correlated ceRNA network	miR-424,	LATS1/2	Hippo	CD44	Stemness markers, sphere formation, chemoresistance	Inhibit	[82]
		miR-374a,						
		miR-590–3p,						
		miR-448,						
		miR-15a,						
	HOTAIR	miR-34a	SOX2	p53/p21, Wnt/β-catenin	c-Myc, Nanog, SOX2, OCT4, Bmi1, β-catenin	Stemness markers, sphere formation, migration	Promote	[83]
	HOTAIR	miR-7	SETDB1, STAT3	STAT3	CD44, CD326, ALDH	Stemness markers, migration and invasion	Inhibit	[84]
	HOTTIP	miR-148a-3p	WNT1	Wnt/β-catenin	OCT4, SOX2, CD44	Stemness markers, sphere formation	Promote	[85]
	CDR1as	miR-7	LKB1, AMPK, mTOR	Autophagy	CD44	Stemness markers	Promote	[86]
	CircNOLC1	miR-365a-3p	STAT3	STAT3	ABCG2, c-Myc, Bmi1, SOX2, Vimentin	Stemness markers, sphere formation, migration, invasion	Promote	[87]
	CircVRK1	miR-153	–	TGF-β, mTOR	CD44, NANOG, SOX2, OCT4	Stemness markers, sphere formation	Inhibit	[88]
	Circ_002178	miR-1258	KDM7A	–	ALDH1, CD44, NANOG, OCT4	Stemness markers, sphere formation, migration, invasion	Promote	[89]
Colorectal	OCT4B	miR-145,	–	–	OCT4A, SOX2	Stemness markers	Promote	[90]
		miR-335, miR-20a,						
		miR-20 b,						
		miR-106a, miR-106 b						
	HOTAIR	miR-211–5p	FLT-1	–	CD133, CD44, Nanog, SOX2, OCT4	Stemness markers, sphere formation, migration, invasion	Promote	[91]
	MEG3	miR-708	SOCS3	JAK/STAT3	LGR5	Stemness markers	Inhibit	[92]
	LINC-ROR	miR-145	–	–	CD44, CD133, OCT4, SOX2, NANOG	Stemness markers	Promote	[93]
	LINC00657	miR-203a	ZEB1, ZEB2, Snail2	–	CD44, CD133	Stemness markers, invasion	Promote	[94]
	LOCCS (LINC01567)	miR-93	MSI1, HDAC8,	–	CD44, CD133, CD166, Oct-4, ABCG2, SOX2, KLF4	Stemness markers, invasion, migration, colony formation, chemoresistance, xenograft	Promote	[95]
			TLE4					
	circAGFG1	miR-4262, miR-185–5p	YY1, CTNNB1	Wnt/β-catenin	CD44, SOX2, OCT4, Nanog	Stemness markers, colony formation, sphere formation, xenograft	Promote	[96]
	circ_001680	miR-340	BMI1	–	CD44, CD133, SOX2	Stemness markers, sphere formation, colony formation, migration	Promote	[97]
	circ_0066631, circ_0082096	miR-140–3p, miR-224, miR-382, miR-548c-3p, miR-579	ACVR1C/ALK7, FZD3, IL6ST/GP130, SKIL/SNON, SMAD2, WNT5A	Wnt/β-catenin, TGF-β/SMAD, Activin/Nodal, GP130/STAT	CD133, CD44, ALDH1	Stemness markers, colony formation, migration, invasion, EMT, chemoresistance	Promote	[98]
Glioma	XIST	miR-152	KLF4	MEK1/2 and PI3K	CD133, Nestin	Stemness markers, proliferation, migration, invasion	Promote	[99,100]
	GAS5	miR-196a-5p	FOXO1	–	CD133, Nestin	Stemness markers, proliferation, migration, invasion	Inhibit	[101]
	UCA1	miR-1,	Slug	TGF-β	Nanog, ALDH1	Stemness markers, sphere formation	Promote	[102]
		miR-203a						
	MYOSLID: 11	miR-149–3p	PXN	–	–	–	Promote	[103]
	SOX2OT	miR-194–5p, miR-122	TDGF-1	JAK/STAT	CD133	Migration, invasion	Promote	[104]
	LINC02381	miR-128, miR-150	IGF1R, TrkC, PI3K, RAS/MAPK	IGF1R, PI3K, RAS/MAPK	–	–	Promote	[105]
	circPTN	miR-145–5p, miR-330–5p	SOX9, ITGA5	–	SOX2, SOX9, CD133, Nestin	Sphere formation, stemness markers	Promote	[106]
	circEPHB4	miR-637	SOX10	–	Nestin, OCT4, Nanog, CD133, CD44	Stemness markers, sphere formation	Promote	[107]
Pancreatic	MALAT1	miR-200c, miR-145	SOX2	Wnt/β-catenin	OCT4, Nanog, SOX2, Bmi1, β-catenin, c-Myc	Stemness markers, sphere formation, migration, chemoresistance	Promote	[108]
	SOX2OT	miR-200	–	–	SOX2	Stemness markers	Promote	[109]
	LINC-ROR	let-7 family,	–	–	OCT4, SOX2, NANOG, ALDH1, CD133, CD24, CD44	Stemness markers, sphere formation, proliferation, migration, invasion	Promote	[110,111]
		miR-93–5p, miR-145–3p, miR-320a,						
		miR-320 b						
	LINC-DYNC2H1-4	miR-145	ZEB1, MMP3	–	LIN28, Nanog, SOX2, OCT4, vimentin	Stemness markers, sphere formation, migration, invasion, EMT	Promote	[112]
	AFAP1-AS1	miR-384	ACVR1	–	CD24, CD44, OCT4, CD133, ABCG2, Nestin, CK19	Stemness markers, sphere formation, colony formation, migration, invasion,	Promote	[113]
	GAS5	miR-221	SOCS3	–	OCT4, CD133, Nanog, SOX2	stemness markers, xenograft, metastasis, migration, proliferation	Inhibit	[114]
	FOXD1-AS1	miR-570–3p	osteopontin/SPP1	–	CD133	stemness markers, sphere formation, xenograft	Promote	[115]
	Circ_0092314	miR-671	S100P	AKT	CD133, CD44	EMT, invasion, stemness markers	Promote	[116]
Lung	MEG3	miR-650	SLC34A2	–	OCT4, CD133, CD44	Stemness markers, sphere formation, migration, invasion	Inhibit	[117]
	C8orf34-as1	miR-671–5p	MFAP4	–	OCT4, NANOG	Stemness markers, sphere formation, xenograft, invasion	Inhibit	[118]
	DGCR5	miR-330–5p	CD44	–	CD44, SOX2, Nanog, OCT4	Stemness markers, sphere formation,	Promote	[119]
	*circ_0003222*	miR-527	PHF21B	TGF-β/SMAD	CD44, CD133, OCT4, SOX2	Stemness markers, drug resistance, sphere formation, migration, invasion	Promote	[120]
	*circPOLA2*	miR-326	GNB1	–	Nanog, OCT4, ALDH1	Stemness markers, sphere formation	Promote	[121]
	PKMYT1AR	miR-485–5p	PKMYT1	Wnt/β-catenin	CD44, SOX2, OCT4, Nanog, ALDH1	Stemness markers, sphere formation, proliferation, migration	Promote	[122]
	ROLLCSC	miR-5623–3p, miR-217–5p	–	lipid metabolism	–	metastasis	Promote	[123]
Liver	CD44 3′UTR	miR-34a-5p,	ULBP2	–	OCT4, SOX2, KLF4, c-Myc	NK sensitivity	Inhibit	[124]
		miR-373–3p,						
		miR-520c-3p						
	MALAT1	miR-375	YAP1	Hippo	c-Myc, SOX2, OCT4	Stemness markers, sphere formation	Promote	[125]
	circMALAT1	miR-6887–3p	PAX5, JAK2	JAK2/STAT3	–	Self-renewal	Promote	[126]
	circZKSCAN1	FMRP RNA	CCAR1	Wnt/β-catenin	EpCAM, SOX2, OCT4, c-Myc	Stemness markers, sphere formation	Inhibit	[127]
	RUNX1-IT1	miR-632	GSK-3β	Wnt/β-catenin	ALDH, CD44, SOX2, OCT4, Nanog,	Stemness markers, sphere formation, proliferation, invasion	Inhibit	[128]
Bladder	XIST	miR-200c	–	–	CD133, CD44, KLF4, OCT-4, ABCG2, vimentin, ZEB1/2	Stemness markers, sphere formation, EMT	Promote	[129]
	Circ_103809	miR-511	–	–	integrin α3, CD44	Stemness markers, sphere formation, migration, invasion	Promote	[130]
	*CircSETD3*	miR-641	PTEN	–	N-cadherin, vimentin, ALDH1, OCT4, CD133, CD44	Stemness markers, sphere formation, migration, EMT	Inhibit	[131]
	Circ_0058063	miR-335–5p	B2M	–	SOX2, OCT4, NANOG	Stemness markers, chemoresistance, xenograft, proliferation	Promote	[132]
	SOX2OT	miR-200c	SOX2	–	CD44, ALDH1, N-cadherin, vimentin	Stemness markers, sphere formation, proliferation, migration, invasion, xenograft	Promote	[133]
	HOXA-AS2	miR-125 b	Smad2	TGF-β/SMAD	ALDH1A1, CD44, KLF4, OCT4, HMGA2	Stemness markers, sphere formation, migration, invasion	Promote	[134]
	KCNMB2-AS1	miR-3194–3p	SMAD5	–	CD133, Nanog, OCT14, SOX2, ALDH1	Stemness markers, sphere formation, invasion	Promote	[135]
Gastric	MACC1-AS1	miR-145–5p	CPT1, ACS	TGF-β/SMAD, FAO	CD44, CD133, OCT4, SOX2, LIN28	Stemness markers, chemoresistance	Promote	[136]
	circFAM73A	miR-490–3p	HMGA2	Wnt/β-catenin	CD44, OCT4, SOX2,	Stemness markers, migration, chemoresistance	Promote	[137]
					Nanog			
	HCP5	miR-3619–5p	PPARGC1A	FAO, AMPK	SOX2, OCT4, LIN28, CD133, CD44	Stemness markers, sphere formation, colony formation, proliferation, chemoresistance	Promote	[138]
Prostate	LINC-ROR	miR-145	OCT4	–	OCT4, SOX2, CD44, CD133	Stemness markers, invasion, chemoresistance	Promote	[139]
Ovarian	E2F6	miR-193a	c-KIT	–	c-KIT	Stemness markers, sphere formation, invasion, chemoresistance	Promote	[140]
Laryngeal squamous cell	hg19-circ-0005033	miR-4521, miR-339–5p	STAT5A	–	CD44, CD133	Migration, invasion, chemoresistance	Promote	[141]
Skin	Circ_008913	miR-889	DAB2IP, ZEB1	–	k5, CD34	Stemness markers, sphere formation, migration, invasion	Inhibit	[142]
	LHFPL3-AS1	miR-181a-5p	BCL2	–	ALDH1, Klf4, Nanog, OCT4, SOX2	Stemness markers, sphere formation, proliferation	Promote	[143]
Osteosarcoma	DANCR	miR-33a-5p	AXL	PI3K-Akt	CD44, CD133, CD90, SOX2	Stemness markers, sphere formation, migration, invasion	Promote	[144]
Thyroid	DOCK9-AS2	miR-1972	CTNNB1	Wnt/β-catenin	N-cadherin, CD133, Nanog, OCT4, SOX2, ALDH1A1, c-Myc	Stemness markers, sphere formation, proliferation, invasion, migration, xenograft	Promote	[145]

## 4 Breast cancer

LncRNA *H19* seems implicated in proliferation, differentiation, EMT, and stemness, implying that it plays a role in tumorigenesis and progression [146,147]. *H19* has recently been demonstrated to repress the *P53* protein, implying that *H19* plays a role in carcinogenesis [148]. At the time of implantation, *H19* is activated in extraembryonic cells; however, after birth, its expression in all tissues significantly declines [149]. *H19* enhances sphere-forming capacity while its deletion decreases colony-forming ability [150]. *H19* acts as an endogenous sponge for the tumor suppressor let-7 to regulate cancer metastasis [151]. Compared to surrounding tissues, *let-7* expression is lower in breast tumor tissues. On the other hand, *H19* overexpression or reduction did not affect cell proliferation in breast cancer cells, implying that *H19*-regulated colony formation, spheroid formation, and tumor-initiating activities correlated to self-renewal. The stemness maintenance of breast CSCs via sponging the tumor suppressor *let7* miRNA is one of the ceRNA activities of *H19*. In this line, Peng et al. found that *H19* is overexpressed in breast cancer cells and, by acting as a ceRNA to inhibit the synthesis of miR-let7, promotes the expression of *LIN2*8 mRNA. Indeed, *H19* protects LIN28 from let-7-mediated degradation [73]. *Pre-let-7* elements have a conserved terminal loop that LIN28A binds to it. The LIN28 blocking *let-7* synthesis and repressed let-7 miRNA target genes (*RAS*, *MYC*, and high mobility group AT-Hook 2 [*HMGA2*]), is critical for CSC maintenance [[152], [153], [154]]. Furthermore, via a feedback loop, LIN28 induction can diminish *let-7* expression even further, as well as *H19* being repressed by *let-7*. Surprisingly, increased *H19* expression significantly boosted breast CSCs characteristics such as migration, colony formation, self-renewal, and sphere-forming capacity [14].

Glycolysis, a crucial step in glucose metabolism, contributes to maintaining CSC during hypoxia and nutrient deprivation [155]. According to previous research, CSCs have a higher glycolytic phenotype [156]. To provide energy, pyruvate dehydrogenase kinase 1 (PDK1) prevents pyruvate oxidation through the tricarboxylic acid cycle [157]. PDK1 is a crucial glycolytic enzyme that is correlated to tumor growth, metastasis, and a poor prognosis [158]. By focusing on PDK1, the LIN28 A/B, and let-7g axis modulates the Warburg effect to stimulate tumor proliferation in a way that is independent of the hypoxia-inducible factor (HIF-1) [159]. Recent research has revealed that the oncoprotein HIF-1α directly targets the protein PDK1 [160]. Peng et al. revealed that the activation of glycolysis by PDK1 during hypoxic conditions is necessary for breast CSC reprogramming. They found PDK1 as an H19 downstream target. They demonstrated the mechanism through which the H19/let-7/HIF-1α signaling axis increases PDK1 [74].

LUCAT1 (lung cancer-associated transcript 1) was identified as the target by large-scale microarray screening for the differentially expressed lncRNAs between breast cancer cells and breast CSCs based on prior findings. They evaluated the expression of LUCAT1 in 151 breast cancer specimens and documented the relationship between LUCAT1 and clinical pathological variables and breast cancer prognosis [75]. mir-5582–3p caused tumor cells to undergo cell cycle arrest and apoptosis, and its expression in CRC tissues was noticeably lower than adjacent normal tissues, thus having an anti-cancer effect [161]. mir-5582–3p was a unique suppressor miR that specifically targeted the TCF7L2 (transcription factor 7-like 2) gene to inhibit the stemness of breast cancer cells and breast CSCs [75]. Breast CSC self-renewal is known to be regulated by the Wnt/β-catenin pathway [162]. TCF7L2 is a crucial component of the TCF7L2/β-catenin complex, which triggered the transcription of downstream Wnt pathway proteins [163]. TCF7L2 plays a crucial role in proliferation, metabolism, cell differentiation, stress reactions, and cell death [164]. As a miR-5582–3p sponge, LUCAT1 targeted TCF7L2, activated the Wnt/β-catenin pathway, and promoted breast CSC self-renewal and breast cancer cell growth [75].

Another type of lncRNA known as sprouty RTK signaling antagonist 4-intronic transcript 1 (*SPRY4-IT1*) is produced from an intron region of the SPRY4 gene [165]. Breast cancer cells have a high level of *SPRY4-IT1*, and knocking it down greatly reduces cell proliferation [166,167]. Song et al. investigated *SPRY4-IT1*'s role on breast CSCs and its mechanism [76]. They showed that MCF-7 CD44^+^ cells had increased expression of *SPRY4-IT1* than the MCF-7 parental cells, and *SPRY4-IT1* stimulated the proliferation and self-renewal of MCF-7 cells. *SPRY4-IT1* and transcription factor 7-like 2 (TCF7L2) were bound by *miR-6882*, reducing both expressions.

Cell proliferation and self-renewal of stem cell are encouraged by the Wnt/β-catenin signaling pathway [168]. The stemness of breast cancer cells is maintained by activating this pathway [169]. In the Wnt/β-catenin signaling pathway, TCF7L2 is crucial [170]. Therefore, TCF7L2 regulates breast cancer cell proliferation and stemness through the Wnt/β-catenin signaling pathway [171]. According to these findings, *miR-6882* targets *SPRY4-IT1* to promote the breast CSCs stemness through the Wnt/β-catenin signaling pathway, but *miR-6882* targets TCF7L2 to inhibit stemness traits [76].

LncRNA FEZF1-AS1 (FEZ family zinc finger 1 antisense RNA 1) has been found as an oncogenic ncRNA in tumors and is situated at the opposite strand of the gene FEZF1 [172]. Breast cancer tissue had a significant overexpression of FEZF1-AS1. FEZF1-AS1 knockdown decreased the CD44^+^/CD24^-^ ratio, BCSC cells' capacity to form mammospheres, and the expression of stemness factors (Nanog, OCT4, SOX2), indicating that FEZF1-AS1 silencing has a suppressive effect on the stemness of breast cancer. Additionally, FEZF1-AS1 may support the proliferation, migration, invasion, and development of breast CSCs. Breast CSC and breast cancer sphere subpopulations both showed up-regulated levels of FEZF1-AS1 and Nanog protein/mRNA with a positive association. Both FEZF1-AS1 and Nanog mRNA 3′-UTR were negatively correlated targets of miR-30a. By sponging *miR-30a*, *FEZF1-AS1* regulated the expression of the *Nanog* protein and breast CSC development [77].

According to research by Tang et al. colon cancer-associated transcript-1 (CCAT1) is a lncRNA that is significantly expressed in breast cancer tissues as well as breast CSCs and is associated with a poor prognosis. Subsequently, they revealed that LncCCAT1 encourages breast CSC stemness, proliferation, invasion, and migration. Additional mechanistic studies conducted in vitro and in vivo revealed that cytoplasmic lncCCTA1 upregulates TCF4 expression by competitively binding to miR-204/211 and encourages β-catenin translocation into the nucleus by interacting with miR-148a/152 and ANXA2, which in turn activates Wnt/β-catenin signaling, promotes EMT and activates lncCCAT1 transcription [78]. In addition to being associated with tumor development, the Wnt/β-catenin signaling pathway is essential for CSC activities [173].

LINC RNA feedback loops influence fundamental pluripotency factors such as OCT4, SOX2, and C-MYC. So, LINC RNA may be involved in sustaining CSC characteristics [174,175]. *LINC-ROR* (Regulator of Reprogramming) is a typical lncRNA that has been involved in supporting stem cell pluripotency as well as tumor progression in earlier research [79,176]. Extracellular vesicle-mediated transfer of *LINC-ROR* inhibits the sensitivity of CD133^+^ liver CSCs to chemotherapeutic treatments by regulating transforming growth factor (*TGF*) expression [176].

Hou et al. reported that *LINC-ROR* promotes EMT in breast cancer cells via sponge *miR-205* [79]. Moreover, *LINC-ROR* was revealed as an endogenous sponge that inhibited embryonic stem cell (ESCs) differentiation by binding to *miR-145*, leading to maintaining ESC self-renewal [177]. Given that many ESC-related genes are found in CSCs, it's reasonable to assume that *LINC-ROR* has a role in modulating CSC features [178,179].

*LINC00511* functions as an oncogene by attaching to the histone methyltransferase, enhancer of zeste homolog 2 (EZH2), and inhibiting p57 in non-small cell lung cancer (NSCLC) [180]. Breast cancer cells had higher *LINC00511* expression, which may be associated with proliferation, invasion, and poor prognosis [181,182]. *LINC00511* has a role in the stemness of breast cancer cells since it stimulates the sphere-formation and the expression of stem factors such as Nanog, SOX2, and OCT4 and facilitates CSC features. *LINC00511* acts as a miR-185–3p sponge and targets the transcription factor E2F1. E2F1 interacts with the Nanog promoter to induce its expression. For the stemness and tumorigenesis of breast cancer, the *LINC0*0511/miR*-185-3p*/E2F1/Nanog axis may possess therapeutic potential [80].

LINC00589, also known as TSLNC8, prevents the growth, invasion, and metastasis of glioma, non-small cell lung cancer, and hepatocellular carcinoma [183]. As demonstrated by Bai et al., trastuzumab-resistant breast cancer has a downregulated expression of LINC00589 while its expression reduced trastuzumab resistance, multidrug resistance (MDR), and CSC-like characteristics of HER2-positive breast cancer. Additional mechanistic experiments show that LINC00589 functions as a ceRNA to control the expression of discs large homolog 5 (DLG5) and PR/SET domain 16 (PRDM16) by interacting with miR-100 and miR-452 [81]. By preventing TAZ expression, DLG5 is thought to reduce breast CSC-like features and increase tamoxifen sensitivity [184]. However, PRDM16, a zinc finger transcription factor that targets the EMT, inhibits lung adenocarcinoma metastasis and is related to patient survival [185].

Yes-associated protein (YAP)/transcriptional co-activator with PDZ-binding motif (TAZ), which mediates the crucial role in the Hippo pathway, is known to be a stemness factor in the formation of breast CSCs [186]. YAP1 has been associated with cancer cell proliferation, EMT, chemoresistance, and suppresses cell apoptosis [187]. Upregulation of YAP1 may result in CSC characteristics such as sphere formation and self-renewal [188,189]. The role of YAP1 in acquiring CSC characteristics is mediated by the upregulation of ESC factors such as *OCT4*, *SOX2*, and *NANOG* [190,191]. Also, TAZ was found to induce CSC-like characteristics in breast cancer cells in a previous study, and in numerous types of stem cells, YAP/TAZ are referred to as “stemness factors” [192]. Furthermore, actin remodeling factors could regulate YAP/TAZ activity [193].

The Hippo signaling pathway is a kinase cascade including mammalian STE20-like protein kinase 1 (MST1) and large tumor suppressor 1/2 (LATS1/2), which is critical for cell proliferation, death, and organ growth modulation [194,195]. As essential members of the Hippo pathway, LATS1/2 could phosphorylate and inactivate the downstream effectors, YAP/TAZ.

Furthermore, StAR-related lipid transfer domain protein 13 (STARD13), cadherin 5 (CDH5), homeobox D1 (HOXD1), and HOXD10 (termed the STARD13-correlated ceRNA network) have been identified as upstream regulator of YAP/TAZ transcriptional activity. The potential mechanisms by which the STARD13 and other genes of this network inhibit breast cancer metastasis is that 3’ untranslated region (UTRs) of these genes have numerous miRNA binding sites and inhibit these miRNAs [196]. The STARD13-correlated ceRNA network was able to co-regulate each other by competing for numerous shared miRNA binding sites, resulting in the formation of a ceRNA network to suppress breast cancer metastasis and EMT coordinately [82]. Zheng et al. found that the STARD13-related ceRNA network inhibited the development of breast CSCs. The STARD13-correlated ceRNA network enhanced LATS1/2 activity, indicating that this ceRNA network plays a role in regulating the Hippo pathway. Notably, *CDH5*-, *HOXD1*-, and *HOXD10*-3′ UTRs could not act in this pathway without STARD13, implying that STARD13 played an essential role in bridging HOXD1, HOXD10, and CDH5 with LATS1/2 (Hippo cascade). In same regard, the lack of LATS1/2 reduced the inhibitory effects of the STARD13-correlated ceRNA network on EMT features of CSC, indicating that the tumor-suppressive effects of the STARD13-correlated ceRNA network are mediated by LATS1/2 modulation [82].

Human ESC expansion and long-term survival may be facilitated by Rho GTPase/F-actin signaling [197]. On the other hand, STARD13 may act as a Rho GTPase activating protein (GAP) that inhibits Rho GTPases and thus RhoA activity, causing the cytoskeleton to reorganize [198,199]. F-actin and Rho GTPase rearrangements are essential for YAP/TAZ activity, according to previous research [193,200]. Mechanotransduction is intricately linked with cytoskeletal dynamics, and YAP/TAZ responds to mechanical stimuli of the surrounding extracellular matrix, which alerts cells of the need to maintain stem cell properties [188]. By suppressing Rho GTPase/F-actin signaling, the STARD13-correlated ceRNA network could also control YAP/TAZ activity.

The STARD13-correlated ceRNA network might suppress breast CSC characteristics via two different routes (RhoA/F-actin and LATS1/2 signaling), both of which resulted in translocation of YAP/TAZ from the nucleus to the cytoplasm. Their findings attempt to establish novel cooperation and coordination between oncogenic Rho GTPase/F-actin function and the tumor-suppressive LATS1/2 (Hippo pathway) as two synergistic components of the STARD13-correlated ceRNA in modulating breast CSC characteristics. To summarize, STARD13, which is triggered by CDH5, HOXD1, and HOXD10 ceRNAs, stimulates Hippo signaling by acting as a ceRNA for upregulating the LATS1/2 and blocks the Rho GTPase/F-actin pathway, inhibiting YAP/TAZ and suppressing EMT and CSC development in breast cancer [82]. They predicted that verteporfin, a YAP-TEAD binding inhibitor, may be utilized in combination with other treatments to target breast CSCs. It was found that the ceRNA function of STARD13 3′UTR on LATS1/2 is achieved by targeting other miRNAs, including miR-15a, miR-374a, miR-424, miR-448, and miR-5903p.

A lncRNA called *HOTAIR* (Hox transcript antisense intergenic RNA) has been associated with metastasis in a number of cancer types, including colorectal, pancreatic, lung, and breast cancer [201]. By targeting the SOX2 mRNA, *miR-34a* decreases the protein level of SOX2 [202]. *MiR-34a* is believed to directly interact with HOTAIR, and as a result, downregulate its expression [202]. In CSCs enriched from breast cancer cells, expression of HOTAIR is essential for migration, invasion, and self-renewal potential. Similar effects on proliferation were seen after the introduction of *HOTAIR* as well as antago-*miR-34a* into CSCs. Through controlling miR-34a and subsequently SOX2, HOTAIR upregulation in CSCs isolated from breast cancer cells may be essential for maintaining self-renewal potential [83].

The epidermal growth factor receptor (EGFR) and other oncogenes are reduced by miR-7, which also prevents breast CSCs from metastasis to the brain [203]. By targeting SET domain bifurcated 1 (SETDB1), miR-7 overexpression reduced the number of breast CSCs, partially reverted EMT, and restricted the mobility of breast CSCs [84]. SETDB1 is an enzyme that favorably maintains the status of stem cells by methylating histone H3 on lysine 9 (H3K9) [203]. Recent research has identified SETDB1 as a novel oncogene that is overexpressed in breast cancer. According to Zhang et al. the lncRNA HOTAIR and miR-7 were positively associated in breast cancer patients, and miR-7 and HOXD10 expression returned after HOTAIR was knocked down. They showed that miR-7 played a new role in suppressing SETDB1, reducing the number of breast CSCs, and reversing the EMT of breast CSCs [84].

Recent research has shown that inhibiting autophagy lowers breast CSC drug resistance and inhibits tumor formation in these cells [204]. The circRNA cerebellar degeneration-related protein 1 transcript (*CDR1as*) has multiple miR-7 binding sites [205]. In pancreatic cancer, miR-7 may impede autophagy in a harsh microenvironment [206]. It has been demonstrated that *CDR1as* functions as a ceRNA to bind to *miR-7* competitively, inhibiting its activity [207]. In breast CSCs, *CDR1as* overexpression was linked to an upregulation of autophagy, and *CDR1as* competitive suppression of *miR-7* improved the LKB1-AMPK-mTOR signaling and autophagy-associated genes [86].

It has been hypothesized that the STAT3 signaling pathway was crucial for the self-renewal of breast CSCs [208]. STAT3 was the target of miR-365a-3p in breast cancer cells. Their findings demonstrated that in contrast to non-tumor tissues, breast cancer tissues had elevated circNOLC1 expression. The capacity of breast cancer cells to migrate and invade was decreased when circNOLC1 was inhibited. *CircNOLC1* as another ceRNA by sponging *miR-365a-3p* increased STAT3 expression and contributed to the CSCs characteristics of breast cancer [87].

miR-153 decreased the expression of KLF5, which contributed to the maintenance of the stemness of triple-negative breast cancer [209]. *CircVRK1* may be involved in preventing breast CSC stemness by sponging *miR-153*. Additionally, when circVRK1 was silenced, elevated levels of stemness-related markers, such as NANOG, SOX2, and OCT4, were seen globally [88].

An anti-oncogene called miR-1258 decreased in breast cancer tissues, and it was associated with clinical staging and survival [210]. Clinically, the tumor size, survival rate, TNM stage, and lymph node metastasis are all strongly correlated with the high level of circ_002178. It is generally recognized that KDM7A was crucial for the development, growth, and metastasis of malignancies. KDM7A was identified as a tumor promoter in breast cancer, and its low expression inhibited tumor growth. An in vitro study showed that suppressing KDM7A dramatically decreased breast CSC survival and microsphere formation [211]. The expression of circ_002178 was positively associated with the presence of KDM7A in breast cancer patient tumor tissues, however, miR-1258 was oppositely associated. *Circ_002178* was shown to downregulate *miR-1258* levels and decrease *miR-1258*'s suppressive effect on KDM7A, so promotes the stem-like properties of breast cancer cells [89].

In breast CSCs, LncRNA HOXA transcript at the distal tip (HOTTIP) was significantly upregulated. By binding to miR-148a-3p, HOTTIP upregulate WNT1, hence activating the Wnt/β-catenin signaling cascade, which promotes the stemness of breast cancer. It enhanced cell clonogenicity, upregulated the expression of OCT4 and SOX2 (stem cell markers), and downregulated the expression of CK14 and CK18 (differentiation markers) [85].

## 5 Colorectal cancer

By alternative splicing, the human *OCT4* gene can generate three different mRNA isoforms including *OCT4A*, *OCT4B*, and *OCT4B1* [212]. *OCT4B* is introduced as a non-coding RNA among spliced isoforms, regulating *OCT4A* expression in a miRNA-dependent manner. The *OCT4* protein, notably the *OCT4B* isoforms, is expressed at low levels in most cancers [213]. Li et al. have shown that manipulating *OCT4B* expression may change cell proliferation based on its impact on *OCT4A* via competitive binding with miRNAs. In their study, overexpression of miR-20a, miR-20 b, miR-106a, miR-106 b, miR-145, and miR-335, decreased significantly OCT4 expression in the HCT116 cells, which inhibited cell proliferation. So, *OCT4B* controls *OCT4A* expression as anti-apoptotic ceRNA in tumor cells [90].

*HOTAIR* is another lncRNA that acts as ceRNA and promotes CSC properties in colorectal cancer (CRC). This lncRNA can facilitate the expression of Fms-like tyrosine kinase-1 (*Flt-1*) via downregulating *miR-211-5p* expression and activity. Flt-1 is the type 1 receptor for vascular endothelial growth factor A (VEGF-A) and a key modulator of tumor angiogenesis. It is also known as a potential CSC marker in CRC, which increases its expression and causes tumor initiation, progression, migration, and metastasis [91].

Maternally expressed gene 3 (*MEG3*) is another lncRNA that acts as a tumor suppressor in multiple cancers. According to a variety of studies, *MEG3* is involved in the regulation of chemoresistance, cell proliferation, invasion, and migration through the “sponging” of mRNAs or miRNAs, and its depletion is reported to enhance stem-cell-like properties in a variety of cell types, including germline stem cells, mesenchymal stem cells, and lung cancer cells. In a study, S. Zhang et al. introduced *MEG3* as a ceRNA that prevents the proliferation of colonic stem cells [92]. Their results showed that *MEG3* sponges *miR-708* to enhance the expression of suppressor of cytokine signaling 3 (*SOCS3*). By suppressing the signal transducer and activator of transcription 3 (STAT3), which is essential for CRC lymph node metastasis, SOCS3 reduces proliferation, migration, and invasion [214]. During the early stage of CRC formation, it also suppresses the malignant proliferation of CRC stem cells.

In most cancers, *miR-145* is downregulated [[215], [216], [217]]. The function of *LINC-ROR* as a ceRNA that upregulates *OCT4*, *NANOG*, and *SOX2* expression by sponging *miR-145* was also reported by Yan ZY et al. Their results have shown that *miR-145* can inhibit the expression of *LINC-ROR*, *OCT4*, *NANOG*, and *SOX2*. In contrast, *LINC-ROR* via sponging this miRNA significantly increased colon CSC proliferation and decreased the sensitivity to chemotherapy [93].

Previous research suggested that *LINC00657* may function as an oncogene in colon and gastric cancer [218,219]. High *LINC00657* expression in CRC was correlated to metastasis, poor survival, and advanced clinical stage. According to Zhao et al., *LINC00657* was increased in human CRC cells and CSCs [94]. In vitro CSC invasion was inhibited by *LINC00657* knockdown. Furthermore, *LINC00657* functioned as a *miR-203a* ceRNA, counteracting its activity as a tumor suppressor gene and causing the CSC invasion. In CSCs, after transfection with si-*LINC00657*, the expression of the EMT inducers and cell invasion factors including Snail2, ZEB1, and ZEB2 was decreased; however, their expression was reversed after transfection with si-*LINC00657* plus *miR-203a* inhibitor. By interacting with *miR-203a*, which targets ZEB1, ZEB2, and Snail2, *LINC00657* boosted CSCs invasion.

*LINC01567* (also known as *LOCCS*) is a lncRNA that is clearly increased in colon CSCs. According to a study conducted by Yu et al. *LOCCS* knockdown inhibited cell proliferation, invasion, migration, and tumor xenograft formation. Histone deacetylase 8 (HDAC8) and TLE4 genes are the targets of miR-93, which functions as a cancer inhibitor in CSCs [220]. *LOCCS* acts as a ceRNA for *miR-93*. So, inhibition of *miR-93* leads to an increase in the expression of its targets including Musashi-1 (MSI1), HDAC8, and TLE4 on the mRNA and protein levels. Then, this mechanism can significantly reduce the colon CSCs' capacity to colonize and tumorigenicity [95]. The RNA-binding protein MSI1 directly binds to the mRNAs for p21, p27, and p53 and prevents the translation of checkpoint regulators. MSI1 accelerates switching from G0/G1 to S as a result and modifies the Notch and Wnt pathways, as well as the Akt signaling pathway, to regulate the proliferation of CSCs. CSCs proliferation and its markers (OCT4, SOX2, and c-Myc) are decreased through lowering *Notch1* and *MSI1* expression in spheroid culture. However, *p21CIP1*'s expression levels increased. MSI1 is essential for the maintenance of stem cells' capacity for self-renewal. Additionally, malignancies are believed to have an increased level of expression [221,222]. TLE4 is highly expressed in human colon CSCs, which suggests that it may interact with Wnt, NF-κB, or Notch signaling and plays a role in colon CSC proliferation and differentiation [220]. HDAC8 promotes resistance to MEK1/2 inhibition in colon cancer cells by triggering Akt signaling and stimulating tumor cell growth in the absence of MEK/ERK activity [223].

Previous research has demonstrated that the Wnt/β-catenin signaling pathway plays essential regulatory roles in the development of CRC tumors and stemness [75,224]. Therefore, it has been determined that targeting the Wnt/β-catenin signaling pathway may be beneficial for the treatment of CRC [225]. The canonical Wnt signaling pathway's key effector is the CTNNB1 gene, often known as β-catenin. In CRC cell lines, circAGFG1 was highly expressed, and its knockdown dramatically reduced CRC cell proliferation, invasion, migration, and stemness. CircaAGFG1 knockdown decreased the expression of Wnt/β-catenin pathway components and prevented β-catenin from translocating into the nucleus. Additionally, Yin Yang 1 (YY1) acted as a transcription factor to promote CTNNB1 transcription in CRC cells. By directly sponging *miR-4262* and *miR-185-5p*, *circAGFG1* increased *YY1* expression and *CTNNB1* transcription (β-catenin gene) to promote metastasis and stemness in CRC [96].

According to studies, the tumor suppressor gene miR-340 can control the cell cycle, which in turn affects tumor migration and metastasis in a number of tumors, including gastric cancer [226]. Jian et al. demonstrated through bioinformatics analysis that only circ_001680 exhibited a negative connection with miR-340. As a result, they hypothesized that miR-340 and circ_001680 might interact. They showed that circ_001680 could encourage CRC cell migration and proliferation. Circ_001680 was also demonstrated to act as an RNA sponge, which reduced miR-340 expression. Additionally, the miR-340 target gene BMI1's expression could be enhanced by circ_001680 [97]. BMI1 is a positive regulator encourages cancer cells to exhibit stem cell-like characteristics [227]. *Circ_001680* may expand the number of CSCs in CRC by upregulating the *miR-340* target gene *BMI1* [97].

Rengganaten et al. produced CRC cells that possess stemness characteristics using spheroid culture, and then they subjected them to high-throughput genome-wide RNA sequencing targeting circRNAs in order to clarify the regulatory and stemness molecular mechanisms in CRC stem cells. Circ_0082096 and Circ_0066631, two circRNAs that were considerably up-regulated, were found in the spheroid cells. These circRNAs were used to illustrate a network of circRNA_miRNA_mRNA that controls the stemness-mediating pathways. It was predicted that the *circ_0082096* and *circ_0066631* would target and sponge the *miR-140-3p*, *miR-224*, *miR-382*, *miR-548c-3p*, and *miR-579*. In addition, The KEGG analysis uncovered six essential mRNAs that were targeted by the circRNA-miRNA axis in the control of pluripotency and up-regulated in the spheroid cells: ACVR1C/ALK7, FZD3, IL6ST/GP130, SKIL/SNON, SMAD2, and WNT5A. Therefore, these six mRNA targets predominantly engaged in signaling pathways (Wnt/β-catenin, TGF-β/SMAD) in the maintenance of stemness in CRC CSCs [98]. The GP130/Stat signaling pathway includes IL6ST/GP130, which interacts with STAT3 to promote self-renewal and naive pluripotency [228]. The phosphorylation and interaction of SMAD2/3 with SMAD4 to reach the nucleus and activate target genes transcription by ACVRLC/AKT7 and SKIL/SNON, operating via the TGF-β and Activin/Nodal signaling pathways [229]. WNT5a and FZD3 modulate β-catenin in the Wnt/β-catenin pathway to promote differentiation, self-renewal, and EMT [230].

## 6 Glioma

The primary regulator of X chromosome inactivation (XCI), X-inactive specific transcript (*XIST*) sets off a series of events that stabilize the silencing of one X chromosome in somatic cells during the early development of female mammals, leaving just a small number of genes active [231,232]. As a ceRNA, lncRNA *XIST* also contributes to the initiation of tumors and other human disorders development [233]. It has been demonstrated that *XIST* knockdown exhibited tumor-suppressive impacts on glioblastoma CSCs by lowering cell proliferation, migration, and invasion as well as triggering apoptosis via up-regulation of *miR-152*. It was also discovered that *miR-152* and *XIST* are located in the same RISC complex, pointing to a potential direct interaction in glioblastoma CSCs. *XIST* and *miR-152* were both repressed in a reciprocal manner [99].

It has been discovered that KLF4 is a downstream target of *miR-152*, which is responsible for the tumor-suppressive actions of *miR-152* in glioblastoma CSCs. KLF4 might bind to and promote LGALS3, which can bind to oncogenic Ras proteins, activating RAF1 and phosphatidylinositol 3-kinase (PI3K) as a consequence. Nevertheless, miR-152-mediated suppression of KLF4 can restrict the activation of the PI3K and MEK1/2 signal pathways [100]. Inhibitors of the PI3K pathway are being extensively developed as anti-cancer therapies since this pathway is activated in a range of human malignancies [234]. The over-expression of *miR-152* can suppress KLF4 expression by targeting its 3′-UTR [100].

According to previous studies, lncRNA growth arrest-specific transcript 5 (*GAS5*) can inhibit the Wnt/β-catenin signaling pathway, thus preventing invasion, metastasis, and angiogenesis in various tumors. The tumor suppressor gene GAS5 is down-regulated in a variety of cancer types and has been associated with a poor prognosis [235]. By acting as a molecular sponge for miR-196a-5p, GAS5 enhances forkhead box protein O1 (FOXO1) expression while decreasing downstream PI3K/AKT phosphorylation. FOXO1 can control the cell cycle arrest and apoptosis of tumor cells [236,237]. The role of *GAS5* as ceRNA in CSCs also demonstrated by Zhao et al. *MiR-196a-5p* via downregulation of the FOXO1 expression stimulates glioma stem cell proliferation, migration, and invasion [101]. Their data showed that *GAS5* exerted tumor suppressive functions in glioma stem cells via sponging *miR-196a-5p*, thus leading to decreased tumor migration and invasion.

LncRNA urothelial carcinoma-associated 1 (*UCA1*) has been found to stimulate the proliferation, migration, and invasion of cervical cancer cells or glioma cells by modulating the expression of *miR-206*, *miR-122*, and *miR-182* [[238], [239], [240]]. By upregulating zinc finger E-box binding homeobox 1 (ZEB1), *UCA1* could sponge *miR-204-5p* to stimulate glioma cell motility, invasion, and EMT [241]. According to the He et al. study, *UCA1* regulates glycolysis that is conducted by glioblastoma stromal cells and glioma cell invasion [242]. TGF-β, a significant EMT activator, accelerates the invasion and metastasis of NSCLC and enhances the stemness of MiaPaCa-2 pancreatic cancer cells [243,244]. According to Li et al., TGF-β triggered the *UCA1* expression in glioma cells [102]. Moreover, TGF-β promoted EMT and stemness, whereas *UCA1* knockdown inhibited this action. They also found that *UCA1* acted as a ceRNA by binding to *miR-1* and *miR-203a* competitively, increasing the expression of *Slug*, a downstream effector of the TGF-β signaling pathway. In a variety of cancers, including colorectal, renal, lung, esophageal, and head and neck cancer, *miR-203a* functions as a tumor suppressor gene. It inhibits invasion by suppressing its target genes, including ZEB1, ZEB2, and Snail2 expression [218,[245], [246], [247], [248]]. Overexpressing Slug reversed the effects of *UCA1* knockdown on glioma cell EMT and stemness, and their expression showed a positive association in glioma tissues. These findings imply that *UCA1* plays a crucial role in regulating EMT, stemness, and drug resistance, suggesting that it may be a promising target for glioma therapy.

Another study used TCGA to incorporate the ceRNA network into the glioma stem cell differentiation process. Zhao et al. identified lncRNA *MYOSLID: 11* as a ceRNA that regulated the expression of the downstream gene paxillin (*PXN*) by binding to *miR-149-3p* competitively in glioma CSCs. The increased expression of *PXN* strikingly increases tumor cell and stem cell migration and invasion [103].

A lncRNA called SOX2OT has a positive correlation with the growth, invasion, and migration of tumor cells [249,250]. The upregulation of SOX2OT in gliomas was shown by Su et al. By targeting miR-194–5p and miR-122, SOX2OT was able to increase the SOX3 production. Teratocarcinoma-derived growth factor 1 (TDGF-1) is expressed transcriptionally by SOX3 and influences GSC biological activity via the JAK/STAT pathway [104]. The EGF-Cripto FRL gene family includes TDGF-1, which controls early embryonic development and cell differentiation [251]. In many cancers, including glioblastoma, TDGF-1 is highly expressed and is crucial for EMT, tumor invasion, and metastasis [[252], [253], [254]]. The JAK/STAT signaling system can be activated to encourage the proliferation, invasion, and other biological functions of tumor cells.

*LINC02381* functions as a tumor suppressor or oncogene in many cancer types. Nemati et al. indicated that insulin-like growth factor1 receptor (IGF1R) and tropomyosin receptor kinase C (TrkC) receptors as well as their downstream pathways (RAS/MAPK and PI3K), were elevated in gliomas when LINC02381 was overexpressed. *LINC02381* sponges *miR-128* and *miR-150*, increasing the IGF1R signaling pathway to play its oncogenic role. Overexpression of *LINC02381* increased the expression of IGF1R, stemness, and EMT markers accelerated migration and decreased the rate of apoptosis [105].

According to the study by Song et al. [255], several circRNAs were elevated in glioma tissues as compared to healthy brain tissue. Chen et al. chose the circPTN, which is produced from the pleiotrophin (PTN) gene in glioma cell lines. CircPTN enhanced the proportion of S-phase cells and contributed to cell proliferation. They made the hypothesis that circPTN could operate via sponging miR-330–5p and miR-145–5p based on the bioinformatics prediction and confirmed by experimental evidence. The downregulation of SOX9 and ITGA5 caused by miR-145–5p and miR-330–5p could be reversed by circPTN. Through the sponging of miR-145–5p, circPTN stimulated glioma stem cells to tumor sphere formation and stemness marker expression like CD133, Nestin, SOX2, and SOX9 [106].

The receptor tyrosine kinase EPHB4 is up-regulated in gliomas, and the circRNA ephrin type-B receptor 4 (circEPHB4, hsa_circ_0081519) is produced from this receptor [255]. circEPHB4 may interact with miR-637, a tumor suppressor in a variety of human malignancies, according to a bioinformatic study [[256], [257], [258]]. MiR-637 expression was shown to be downregulated in human gliomas, which was correlated with patients' poor prognoses and stimulated glioma development and invasion by activating Akt1 [259]. Additionally, circEPBH4 and SOX10 may both bind to miR-637 in the same areas, according to a bioinformatic study. By increasing Nestin, the oligodendroglial differentiation transcription factor SOX10, which was upregulated in gliomas, enhances the CSCs characteristic in breast cancer [260,261]. The stemness and self-proliferation of glioma cells were promoted by *circEPHB4* via sponging miR-637, which then released its inhibition on SOX10 [107].

## 7 Pancreatic cancer

Metastasis-associated lung adenocarcinoma transcript 1 (*MALAT1*), a universally expressed lncRNA with strong evolutionary conservation, was discovered to be greatly overexpressed in a number of human cancers. It was also found to be correlated with clinical indicators and enhanced tumor cell invasion and metastasis [262]. According to studies, MALAT1 may play a role in the malignancy phenotypes of pancreatic cancer. MALAT1 knockdown was found to block the EMT process and reduce the protein expression of CSC-like markers such as CD24, CD44, and ALDH1 [263]. *MALAT1* knockdown in pancreatic cancer cells inhibited the sphere formation and the expression of self-renewal factors, including *SOX2* and CD133, implying that *MALAT1* may increase pancreatic CSC stemness characteristics by upregulating *SOX2* expression [108].

Exosomal lncRNA *SOX2OT* regulates *SOX2* expression as a ceRNA of *miR-200s*, facilitating PDAC invasion and metastasis [109]. Studies have also shown that *SOX2OT* knockdown with RNA interference can reduce the expression of genes related to stemness, the capacity of tumor spheres to form (both in terms of size and number), and their resistance to docetaxel, which are three of the primary features of tumor spheres in esophageal squamous cancer cells (ESCC) [264]. Furthermore, *SOX2OT* knockdown inhibited the stemness phenotype of bladder CSC, showing its significance in bladder CSC regulation [133]. SOX2 is thought to be a hallmark for undifferentiated cells. SOX2 is crucial in maintaining the pluripotency of ES cells or pluripotent stem (iPS) cells in an undifferentiated state. The stimulation of the Wnt signaling pathway or the EMT pathway is how the SOX2 transcription factor contributes to cell invasion, metastasis, and migration [265]. Overexpression of *SOX2* and related aberrant interacts with various signaling pathways such as unchecked proliferation, apoptosis resistance, altered autophagy, enhanced EMT, and maintenance of CSCs, promoting tumor development, metastasis, and treatment resistance [266].

*LINC-ROR* is a lncRNA that has been proven to play an important function in maintaining the pluripotency of human ESC and compliance with its role in induced pluripotent stem (iPS) cells. Also, it plays essential roles in the progression of tumors, EMT, and stem cell pluripotency maintenance [267,110]. Fu et al. discovered that *LINC-ROR* expression correlates to stemness in pancreatic cancer cells. In vitro, *LINC-ROR* knockdown reduced pancreatic cancer cell proliferation, colony-forming ability, and invasion and impaired pancreatic cancer cell stem-like properties. Also, they revealed that silencing *LINC-ROR* inhibited CSC marker expression, sphere formation, and carcinogenesis. By comparing microarray data, they discovered numerous CSC inhibitory miRNAs, including several members of the let-7 family, *miR-93-5p*, *miR-145-3p*, *miR-320a*, and *miR-320b* were bound to *LINC-ROR* transcript [111]. Notably, *LINC-ROR* demonstrated potential ceRNA activity targeting other tumor-suppressor miRNAs, including *miR-205*, *miR-181a*, *miR-99b*, and *let-7a-5p* [79,268], reducing their effective concentrations [79,177,268], and protect core transcription factors in CSCs [269].

*ROR*, which is upregulated in pancreatic ductal adenocarcinoma (PDAC) tissues, is being used to predict prognosis in patients with pancreatic cancer. By binding to the Nanog mRNA 3′UTR or *ROR*-specific regions in pancreatic CSC, *miR-145* can mediate post-transcriptional silence of its targeted genes. *MiR-145* inhibits the expression of *Nanog* by interacting with this location and causing the Dicer enzyme to cleave *Nanog* mRNA. *ROR* binds a large percentage of *miR-145*, leaving little for suppressing *Nanog* expression. The findings also imply that *ROR* and Nanog are in competition with one another for *miR-145*. One of the crucial aspects for pancreatic CSCs to maintain their high rates of proliferation, invasion, and tumorigenesis is to maintain this “*ROR*-*miR-145*-Nanog” association in balance [110].

In the pancreatic cancer cell line, linc-DYNC2H1-4 was upregulated. It was related to the pancreatic cancer cells' invasion, CSC acquisition, and EMT characteristics. Lin28, Nanog, SOX2, OCT4, ZEB1, and MMP3 were upregulated as a result of linc-DYNC2H1-4's competition with miR-145, which controls EMT and CSCs [112]. MiR-145 is a well-known tumor suppressor [270], that inhibits the expression of ZEB1, a critical regulator of EMT, and embryonic transcription factors, Lin28, Nanog, OCT4, and SOX2 in a variety of cancer models [271,272].

In pancreatic cancer tissues, there was a high expression of actin filament-associated protein 1 antisense RNA 1 (AFAP1-AS1) and activin receptor A type I (ACVR1), but a low expression of miR-384. It was found that when miR-384 was up-regulated, ACVR1 was down-regulated. While AFAP1-AS1 inhibition reduced its capacity to bind to miR-384 in a competitive manner, it increased miR-384 expression and downregulated ACVR1, thereby slowing the growth of pancreatic cancer. It has been demonstrated that miR-384 overexpression or AFAP1-AS1 knockdown reduces pancreatic cancer cells' capacity for tumorigenicity, migration, invasion, self-renewal, and stemness [113].

According to reports, GAS5 acts as a tumor suppressor in several malignancies. According to Liu et al. upregulation of GAS5 inhibited pancreatic cancer cells' proliferation, EMT, migration, resistance to gemcitabine, and stem cell-like characteristics by directly binding to miR-221, inhibiting its expression, and increasing SOCS3 expression [114].

A variety of human cancers have shown downregulation of miR-671 [[273], [274], [275]], which inhibits the EMT abilities of pancreatic adenocarcinoma (PAAD) cells. Human cancers, including PAAD, commonly overexpress the calcium-binding protein S100P [276]. S100P was found to enhance PAAD cells' capacity for invasion and migration through the AKT pathway activation [277]. According to Shen et al., *circ_0092314* was upregulated significantly in PAAD cells and promoted the expression of CD133 and CD44, leading to EMT and CSC features. By sponging miR-671, Circ_0092314 can also upregulate S100P expression, promoting EMT and invasion in PAAD cell [116].

In pancreatic CSCs, Ouyang et al. discovered that the lncRNA FOXD1-AS1 was elevated. FOXD1-AS1 acts as a ceRNA to sponge miR-570–3p and increases osteopontin/secreted phosphoprotein 1 (SPP1) to promote self-renewal and carcinogenesis in pancreatic CSCs. Furthermore, FOXD1-AS1 expression levels were found to be significantly higher in PC cell lines that were resistant to 5-FU [115].

## 8 Lung cancer

In lung cancer tissues that are resistant to chemotherapy, the lncRNA MEG3 is markedly downregulated [278]. The role of *MEG3* as a ceRNA also was reported in lung CSCs. The ability of lung cancer cells to form spheres, express OCT4 and CD133, and develop a stem cell-like state were all boosted when MEG3 was suppressed. Upregulation of *MEG3* promotes the protein level of solute carrier family 34 member 2 (SLC34A2) and curbs migration and invasion in lung cancer cells and lung CSCs by sponging *miR-650* [117]. MiR-650 has been observed to be increased in NSCLC and may function as an oncogene in a variety of malignancies, including human lung adenocarcinoma [279,280]. In the lung metastasis and H1299 subcutaneous tumor model, SLC34A2, a sodium-driven phosphate cotransporter, reduces tumor development and metastatic ability [281,282]. According to Yang et al. SLC34A2 is downregulated in lung cancer cell lines, and its overexpression prevents NSCLC from surviving and invading [283]. Additionally, it has been revealed that SLC34A2 preserves CD147^+^ breast CSCs' stem cell-like properties [284]. Cell migration and invasion was restored by the SLC34A2 inhibition, which was prevented by *miR-650* downregulation. So, *MEG3* as a ceRNA plays an important role in the stem cell-like state of lung cancer cells and inhibits migration and invasion in NSCLC via the *miR-650*/SLC34A2 axis [117].

Han et al. found novel cancer stemness-related ceRNA axis (*C8orf34-as1*/*miR-671-5p*/MFAP4) in lung adenocarcinoma (LUAD) via multiple bioinformatics analyses [118]. Microfibrillar-associated protein 4 (MFAP4) is an extracellular glycoprotein that may be involved in cell adhesion [285], and dysregulation of this protein is reported in a variety of malignant tumors [286]. In prostate and urinary bladder cancer, MFAP4 acts as a tumor suppressor [287,288], and by targeting *MFAP4*, *miR-147b* increased aggressiveness in LUAD cells [289]. Low expression of *MFAP4* was associated with LUAD patients' poor prognosis. Also, it was revealed that lncRNA *C8orf34-as1* is correlated to the prognosis of LUAD patients [290]. The results of Han et al. have shown that the *C8orf34-as1* by binding to *miR-671-5p* acts as an endogenous sponge and abrogating miRNA-induced MFAP4 suppression. This modulatory ceRNA axis associated with the LUAD stemness and invasion [118].

According to Dong et al. lncRNA DGCR5 can encourage the growth of lung adenocarcinomas [291]. They found that DGCR5 was elevated in lung CSCs and that blocking it can prevent NSCLC cells from possessing CSC-like characteristics. They observed that lung CSCs had lower miR-330–5p levels. MiR-330–5p mimics have the ability to block CSC-like features, and research has shown a negative association between DGCR5 and miR-330–5p. In addition, miR-330–5p was predicted to target CD44. Therefore, they observed that *DGCR5* can contribute to NSCLC stemness via regulating CD44 by sponging *miR-330-5p* in vitro [119].

miR-527 is related to the suppression of the EMT and has a suppressive role in NSCLC by inhibiting the TGF-β/SMAD signaling pathway [292]. Tumor tissue had decreased levels of miR-527 expression, which inhibits stemness. Circ_00003222 is derived from the LARP4 gene, which is implicated in RNA stability, cell division, migration, and invasion. Circ_00003222 was overexpressed in NSCLC patients' tumor tissue and inhibits miR-527 [293,294]. miR-527 inhibition reverses the NSCLC cell proliferation and stemness. The regulatory target of miR-527 was PHF21B, and tumor tissues from NSCLC patients have considerably higher levels of PHF21B [120]. PHF21B overexpression has been observed to stimulate cells with CSC-like characteristics in prostate cancer by activating the Wnt/β-catenin signaling pathway [295]. So, *circ_0003222* increases stemness and the development of NSCLC by sponging miR-527 [120].

Acute lymphoblastic leukemia progression and tyrosine kinase inhibitor resistance in leukemia have been associated with the protein subunit beta 1 (GNB1) [296]. A recently discovered circRNA DNA polymerase α2 accessory subunit (circPOLA2 or hsa_circ_0022812), was found by Fan et al. to be overexpressed in lung cancer and to indicate a poor prognosis [121]. Importantly, research on miR-326 has revealed that it inhibits the development of several malignancies, including lung cancer [297,298]. It has been suggested that it controls Cyclin D1 expression during the growth of NSCLC [298]. When compared to adjacent control tissues, the level of miR-326 was significantly lower in lung cancer. miR-326 mediates the inhibition of lung cancer cell stemness by suppressing GNB1, which supported the stemness of lung cancer cells. By sponging miR-326, *circPOLA2* performs the role of a ceRNA and controls GNB1 expression in lung cancer cells [121].

Yin Yang 1 (YY1) factor induces protein kinase, membrane-associated tyrosine/threonine 1 associated lncRNA (PKMYT1AR) in NSCLC, particularly in malignant cells. He et al. demonstrates that PKMYT1AR knockdown reduces the proliferative, migratory, and tumor-forming capacities of tumor cells. They show that miR-485–5p and PKMYT1AR directly interact, diminishing the inhibitory effect on PKMYT1, the downstream oncogenic factor. The PKMYT1AR/miR-485–5p/PKMYT1 axis increases maintenance of CSCs in NSCLC by suppressing β-TrCP1-mediated β-catenin degradation, which then results in increased carcinogenesis [122].

Through the manipulation of lipid metabolism by miR-5623–3p and miR-217–5p, a new lung CSC extracellular vesicles lncRNA ROLLCSC regulates non-stemness cancer cell plasticity. Zhang et al. demonstrate how ROLLCSC is transported to lung cancer cells via CSC-encapsulated extracellular vesicles. There, it functions as a ceRNA for miR-5623–3p and miR-217–5p, promoting lipid metabolism and eventually enhancing lung cancer cells' ability to metastasize [123].

## 9 Liver cancer

UL16 binding protein 2 (ULBP2) is expressed on cancer cells and binds to the natural killer (NK) cell activating ligand, NKG2D, increasing cancer cell sensitivity to NK cell-mediated cytotoxicity [299]. Contrary to most of the previous studies that the tumor-suppressive roles of miR-34a have been mentioned [300,124], Anja Heinemann et al. revealed that miR-34a and miR-34c are adversely related to surface ULBP2, sensitize tumor cells to kill by NK cell [301]. According to Weng et al. ULBP2 and CD44 expression levels were associated, and this had an impact on how susceptible CSCs were to NK cell-mediated cytotoxicity. CSCs express significant levels of surface CD44 and are sensitive to cytotoxicity caused by NK cells. On the other hand, cell proliferation and colony formation are inhibited by CD44's non-coding 3′-UTR, whereas cell adhesion, motility, and invasion are enhanced. Since ULBP2 is a target of miR-34a, CD44 3′UTR interferes with miR-34a′s binding and increases the *ULBP2* expression in liver CSCs, which causes NK cells to kill the CSCs. Their findings suggested that CD44 3′UTR could act as a ceRNA to protect *ULBP2* expression by competing with miR-34a, miR-373, and miR-520c [302].

In another study, *MALAT1* is introduced as highly conserved lncRNAs, which could maintain the stemness of liver CSCs by upregulating YAP1 via sponging miR-375 [125]. *MALAT1* was upregulated in different cancers and has been associated with tumor progression, invasion, and chemoresistance [[303], [304], [305], [306]]. Also, it has been correlated with CSC characteristics (sphere formation and the upregulation of stemness markers) of pancreatic cancer, osteosarcoma, and glioma [108,307,308]. MiR-375 has previously been found to be significantly downregulated in a variety of cancer types, including liver cancer [309]. By targeting a number of crucial oncogenes, miR-375 could reduce liver cancer cell proliferation and migration and overcome chemoresistance [310,311]. In the Hippo signaling system, which is crucial for cell proliferation, apoptosis, controlling organ growth, inducing EMT, and promoting drug resistance, YAP1 plays a significant role [187,195]. The CSC characteristics of self-renewal, sphere formation, invasiveness, and drug resistance may be induced by YAP1 upregulation [188,189]. Zhao et al. have shown that in addition to promoting sphere formation, YAP1 overexpression also increased SOX2 and OCT4 expression. Knockdown of *MALAT1* with small interfering RNA (siRNA) causes reduced expression of *YAP1*, whereas the inhibition of miR-375 can induce *YAP1* overexpression [125].

One of the differentially expressed circRNAs in the CSCs is derived from MALAT1 and known as circ-MALAT1 (circ_0002082). *circMALAT1* increases hepatocellular self-renewal of CSCs by a novel mechanism. By sponging miR-6887–3p, it can not only increase the expression of JAK2, which is favorable for CSC self-renewal but also repress the expression of Paired box 5 (PAX5), which is unfavorable for self-renewal, by impeding mRNA translation at the ribosome. *CircMALAT1* operates as a brake to block the translation of a tumor suppressor, PAX5 mRNA, by binding both the ribosome (IRESs) and PAX5 mRNA. Hepatocellular CSC self-renewal may be facilitated by up-regulation of *circMALAT1*, which sponge miR-6887–3p and increases the activity of JAK2/STAT3 pathway [126].

Fragile X mental retardation protein (FMRP) is an RNA-binding protein (RBP) and, through its target gene, cell cycle, and apoptosis regulator 1 (CCAR1), contributes to the Wnt/β-catenin pathway activation. The Wnt/β-catenin signaling pathway is co-activated by CCAR1, which also increases cell stemness. Zhu et al. revealed that *circZKSCAN1* reduces stemness in HCC by acting as an RBP sponge, competing with the FMRP target gene, CCAR1, and inhibiting the Wnt/β-catenin signaling pathway [127].

Reduced levels of RUNX1's lncRNA intronic transcript 1 (RUNX1-IT1) were found in HCC samples and the gene expression omnibus (GEO) data set and it was associated with a poor prognosis. In vivo, RUNX1-IT1 inhibited the proliferation, metastasis, and stem-like characteristics of HCC cells. In HCC cells, RUNX1-IT1 modulated the WNT/-catenin pathway by directly binding to miR-632 and acting as ceRNA to promote the expression of the miR-632 target gene, GSK-3 [128].

## 10 Bladder cancer

miR-200c can function as a tumor suppressor to regulate the proliferation, EMT, metastasis, and migration of human bladder cancer cells. Using an online tool called miRcode and concentrating on the XIST, Xu et al. predicted lncRNAs with complementary base pairing with miR-200c. By acting as a sponge for miR-200c, XIST controls the biological processes of cells that resemble human bladder CSCs. Therefore miR-200c inhibition by increased expression of XIST can promote self-renewal, clone formation, and the feature of EMT in CSCs [129].

*Circ_103809*, which was found to be the most variable gene among the differentially expressed circRNAs in bladder CSCs, can act as a sponge for miR-511 by binding to the 3′-UTR region of the gene, preventing it from connecting to the target mRNA. It has been demonstrated that *circ_103809* can promote the production of oncospheres, migration, and invasion of bladder CSCs. The tight junction for circRNAs, hedgehog signaling, and cysteine and methionine metabolism were the highly enriched KEGG pathways for differentially expressed circRNAs. Furthermore, the reduction of *circ_103809* greatly reduced bladder cancer's capacity for metastasis and invasion [130].

Through the sponging of miR-421, circSETD3 suppresses the proliferation of cells both in vitro and in vivo in HCC [312]. CircSETD3 has dramatically downregulated in bladder urothelial carcinoma (BLCA), and it had a negative correlation with lymphatic metastasis, cell migration, proliferation, and stemness maintenance. It inhibits bladder CSC abilities by sponging miR-641 to upregulate phosphatase and tensin homolog (PTEN) [131]. PTEN is a well-known tumor suppressor that prevents BLCA cells from proliferating, migrating, invading, and undergoing EMT [313,314]. Additionally, it has been observed that PTEN downregulation suppresses breast cancer stem cell characteristics [315].

Circ_0058063 was discovered by Sun et al. to be considerably overexpressed in tissue and cells that were cisplatin-resistant. They found that high levels of circ_0058063 expression are related to poor prognosis and that circ_0058063 silencing inhibits proliferation and stem cell properties while increasing cisplatin sensitivity. Additionally, they revealed that circ_0058063 increased the resistance of bladder cancer cells to cisplatin by sponging miR-335–5p to favorably regulate the expression of beta-2-microglobulin (B2M) [132].

A recently discovered lncRNA called SOX2 overlapping transcript (SOX2OT) has a role in the growth of cancer and CSCs and may serve as a biomarker. Increased SOX2OT expression was strongly associated with a poor prognosis and was highly expressed in bladder cancer. Additional research showed that the stemness trait of bladder CSCs was reduced by SOX2OT knockdown. Zhan et al. discovered that SOX2OT sponge miR-200c to favorably regulates SOX2 expression [133].

According to reports, long non-coding RNA HOXA cluster antisense RNA 2 (HOXA-AS2) acts as an oncogene in a variety of cancers. According to Wang et al., HOXA-AS2 is overexpressed in both bladder cancer cells and clinical bladder tumors. Additionally, this increase showed a favorable association with the expression of the CSC marker OCT4. This study also revealed that miR-125 b is a downstream target of HOXA-AS2, that HOXA-AS2 downregulates miR-125 b, and that interactions between HOXA-AS2, miR-125 b, and Smad2 are important for HOXA-AS2's functional role in mediating bladder cancer cells' stemness, migration, and invasion [134].

The elevated level of the lncRNA KCNMB2-AS1 in bladder cancer has been reported by Chen et al. In vitro, KCNMB2-AS1 knockdown reduced the capacity of cancer cells to proliferate, metastasize, and retain their stemness. Next, they discovered that SAMD5 was a downstream target of miR-3194–3p and that KCNMB2-AS1 might interact with it [135].

## 11 Gastric cancer

Fatty acids (FAs) are important energy resources for cancer cells through FA oxidation (FAO), which has been found to be required for cancer cell survival and growth [316]. According to recent reports, FAO has no impact on non-stem cancer cells but may promote breast CSC self-renewal and therapy resistance [208]. By increasing ATP production, metabolic reprogramming toward FAO may regulate chemoresistance [317]. He et al. discovered that inhibiting the FAO rate-limiting enzyme carnitine palmitoyltransferase 1 (CPT1) reduced stemness gene expression and resistance to 5-Florouracil and oxaliplatin [136]. On the other hand, FAO is a significant source of NADPH (nicotinamide adenine dinucleotide phosphate) [316]. Suppression of FAO led to an increase in intracellular reactive oxygen species (ROS), a decrease in NADPH, and ultimately the death of cancer cells [318]. Low ROS levels are characteristic of CSCs, which is essential for maintaining their stemness and capacity for self-renewal [33,319]. Therefore, increasing antioxidant capacity to maintain low ROS could support GC cells' stemness and chemoresistance. By producing antioxidants, lncRNA MACC1-AS1 maintains low ROS [320]. Mechanically, mesenchymal stem cells (MSCs) released TGF-1, which activated SMAD2/3 through TGF- receptors, stimulated the expression of the lncRNA MACC1-AS1 in gastric cancer cells, and boosted FAO-dependent chemoresistance and stemness by inhibiting miR-145–5p [136]. Drug resistance is inhibited by miR-145–5p, which is bound to the 3′UTR of SOX2 mRNA and affects the antitumor response in glioblastoma [321].

circFAM73A, whose expression is considerably elevated in gastric cancer, was discovered by Xia et al. by analyzing GEO databases. After doing a bioinformatic analysis, they screened the TCGA downregulated miRNAs and discovered that miR-490–3p can bind to circFAM73A. The majority of DNA-binding proteins were initially found to be high mobility group (HMG) proteins in embryonic stem cells [137]. In normal mature tissues, HMGA2 expression is especially low and becomes increasingly constrained during fetal development [322]. Interesting research has revealed that HMGA2 is re-expressed during oncogenesis in a number of human cancers, including gastric cancer, where high HMGA2 expression is correlated with sphere formation and lymph node metastasis [[323], [324], [325]]. The downstream target of miR-490–3p is HMGA2, and *circFAM73A* acts as a miR-490–3p sponge. By sponging miR-490–3p and upregulating HMGA2 expression, *circFAM73A* enhances stem cell-like characteristics in gastric cancer. On the other hand, it has previously been established that HNRNPL encourages the generation of circRNA in prostate cancer [326]. It has also been established that HNRNPL binds to the flanking introns of circFAM73A and plays a role in the formation of circFAM73A. *circFAM73A* promotes HNRNPK recruitment, improves the interaction between β-catenin and HNRNPK, and promotes β-catenin stability, consequently promoting CSC-like characteristics in gastric cancer [137].

Wu et al. wanted to understand how MSC-induced lncRNA modulated in gastric cancer. Based on GEO data, dysregulated lncRNAs in gastric cancer were investigated. Results showed that MSC co-culture promoted LncRNA histocompatibility leukocyte antigen complex P5 (HCP5) in gastric cancer cells, resulting in stemness and therapeutic resistance. The FAO was prompted to support chemoresistance and stemness of gastric cancer cells when HCP5 sequestered miR-3619–5p and upregulated peroxisome proliferator activated receptor gamma coactivator 1 alpha (PPARGC1A), enhancing the transcription complex PPAR coactivator 1 (PGC1)/CCAAT enhancer binding protein (CEBPB), and transcriptionally inducing CPT1 [138].

## 12 Prostate cancer

The expression of *LINC-ROR* was found to be significantly associated with human prostate CSCs proliferation. In contrast, the expression of miR-145 was found to be adversely associated with prostate CSCs proliferation. Liu et al. found that curcumin inhibits prostate CSCs proliferation, invasion, and tumorigenicity through ceRNA actions of miR-145 and *LINC-ROR*. *OCT4* and *LINC-ROR* are both suppressed by miR-145. In prostate CSCs, decreasing the expression of endogenous *LINC-ROR* substantially increased the concentration of miR-145, whereas miR-145 inhibits cell proliferation via reducing *OCT4* expression. The expression of *OCT4* and *LINC-ROR* was balanced, allowing prostate CSCs to maintain the expression of cell cycle kinases and progress through the cell cycle, increasing their proliferation and invasion [139].

## 13 Ovarian cancer

The role of ceRNA networks in cancer stemness and progression was reported in ovarian cancer by Cheng et al. [140]. E2F transcription factor 6 (E2F6) is upregulated in ovarian cancer when estrogen (E2) binds to the estrogen receptor (ER). Upregulated *E2F6* mRNA, in turn, upregulates the oncogene *c- KIT*, a promotor of cancer stemness. Overall, *E2F6,* via competitive inhibition of miR-193 binding, acts as a ceRNA to increase the expression of the *c-KIT*, thus advancing tumor progression.

## 14 Laryngeal squamous cell carcinoma

In contrast to CD133^+^ or CD44^+^ stem cells and CD133-CD44^−^stem cells, Wu et al. showed that laryngeal CD133^+^CD44^+^ CSCs exhibit more aggressive malignant characteristics. A whole-transcriptome analysis of CD133^+^CD144^+^ CSC derived from human laryngeal squamous cell carcinoma cells revealed that *hg19-circ_0005033* acts as a ceRNA to upregulate miR-4521 target mRNAs and increases proliferation, migration, invasion, and chemoresistance. Their findings showed that miR-339–5p and miR-4521 interact with hg19_circ_0005033 and target STAT5A [141]. In particular, STAT5A promotes the EMT and stem-like cell characteristics in prostate cancer [327].

## 15 Skin cancer

Diverse cancer types have decreased levels of disabled-2 interacting protein (DAB2IP), a Ras-GTPase activating protein that inhibits Ras-dependent mitogenic signals [328,329]. Its functions include preventing EMT and modifying signal cascades correlated with cell proliferation [330]. DAB2IP also activates ZEB1 gene expression by inhibiting the PI3K-Akt-mTOR signaling pathway, which results in enhanced CSC features [331]. Arsenite exposure resulted in the acquisition of CSC-like features in transformed immortalized human keratinocytes, as seen by the enhanced expression of CD34 and k5. K5 expression is a hallmark of undifferentiated skin stem cells or CSCs, while CD34 is particularly expressed in keratinocyte stem cells in skin CSCs. Circ008913 may bind to miR-889, according to bioinformatics techniques used to anticipate the miRNAs that interact with it. Through its target, DAB2IP, miR-889 encourages the growth of carcinomas in esophageal squamous cells [332]. Circ008913 levels are reduced due to arsenite, which also acts as a sponge for miR-889, deregulates DAB2IP by elevating miR-889 and ZEB1 levels, which is necessary for the arsenite-induced acquisition of CSC-like characteristics [142].

An essential molecular characteristic of cancer is the inhibition of apoptosis by B-cell lymphoma-2 (Bcl-2). According to research by Zhang et al. the lncRNA LHFPL3-AS1-long increased the BCL2 protein and participated in the carcinogenesis of melanoma stem cells. LHFPL3-AS1-long directly interacted with miR-181a-5p to prevent Bcl-2's mRNA from being degraded, inhibiting melanoma CSCs apoptosis. In melanoma stem cells, the alternative splicing of the LHFPL3-AS1 transcript was controlled by the splicing factor polypyrimidine tract binding protein 1 (PTBP1). This resulted in the formation of LHFPL3-AS1-long. Expressions of PTBP1 and LHFPL3-AS1 were considerably increased in melanoma patients [143].

## 16 Osteosarcoma

Upregulation of the lncRNA differentiation antagonizing nonprotein coding RNA (DANCR) enhanced osteosarcoma cell proliferation, migration, invasion, and lung metastasis. Additionally, DANCR increased the expression of proteins downstream of the AXL-Akt pathway and amplified the receptor tyrosine kinase AXL by competitively binding to miR-33a-5p [144]. AXL modulates EMT, chemoresistance, and tumor cell self-renewal [333]. By upregulating AXL through competitively binding to miR-33a-5p, DANCR improves CSC function. This function is successively carried out via the PI3K-Akt signaling pathway [144].

## 17 Thyroid carcinoma

Based on the findings of differentially expressed lncRNAs through bioinformatics databases, the exosomal lncRNA DOCK9 antisense RNA2 (DOCK9-AS2) was found to be elevated in plasma exosomes of patients with papillary thyroid cancer (PTC). In PTC cells, DOCK9-AS2 knockdown decreased proliferation, EMT, stemness, migration, and invasion. Exosomal DOCK9-AS2, which was created from PTC-CSCs, increased the stemness of PTC cells. In PTC cells, the Wnt/β-catenin pathway was activated mechanistically by the interaction of DOCK9-AS2 with SP1 to promote catenin beta 1 (CTNNB1) transcription and sponge miR-1972 to upregulate CTNNB1 [145].

## 18 Discussion

There are still few trustworthy biomarkers or efficient therapeutic techniques for routine clinical practice in cancer, despite the fact that there have been countless attempts over the past few years to increase the efficacy of chemotherapy or surgery in cancer patients. Growing data suggest that ncRNAs may be employed as innovative potential therapeutic targets for treating a variety of malignancies because of their high capacity to control cancer cell proliferation, EMT, invasion, metastasis, apoptosis, and response to chemotherapy.

Cancer cells' ability to self-renew and exhibit stem-like characteristics can lead to their recurrence and metastasis. As a result, further therapies targeting CSCs for cancer treatment are urgently needed, particularly for that whit a poorly differentiated “stem/progenitor” cell phenotype. The successful development of novel treatment strategies that target CSCs has the potential to improve cancer patient outcomes. The current study offers the groundwork for the establishment of novel therapeutic approaches to eliminate CSCs that could be hiding in remaining malignant lesions. On the other hand, considering the comprehensive impacts of the ceRNA network on different pathways, a cancer treatment strategy driving the ceRNA network might be proposed.

CSCs have been the subject of numerous clinical trials, which point to a bright future for cancer treatment. However, there are also a number of challenges that must be resolved. It is still not completely clear which CSCs inhibition or activation is beneficial to the cancer patient [334]. Many CSCs, in particular cancers and their environmental elements, have not been well characterized [335,336]. It is urgently necessary to identify CSCs and isolate them by focusing on multiple markers. The low frequency of these cells in many cancers necessitates powerful purification techniques which are not yet widely accessible [337]. Since normal stem cells have many similar characteristics to CSCs, it is clear that choosing a molecule or signaling pathway that specifically inhibits CSCs, not normal stem cells, is of great importance. Another issue that should be considered is the behavioral differences between the CSCs of different tumor stages as well as different tissues [338]. In addition, there are differences between CSCs in the original tumor environment and in vitro conditions. CSCs have a niche in which they can survive, although most current studies use isolated CSCs without a microenvironment [339]. Because the majority of CSC research is conducted in immuno-deficient mice, these models do not accurately represent the molecular complexity of the tumor microenvironment [340].

CeRNA networks, a novel form of RNA-to-RNA interaction, have been discovered, and it has been shown that these interactions are essential for the development of malignancies. In addition to serving as predictive and diagnostic markers, they may also function as therapeutic targets. Numerous RNAs function as ceRNAs in CSCs, targeting miRNAs to regulate the expression of pluripotency factors, CSC markers, and modifying the stemness process. Given that CSC have special characteristics that increase resistance to anti-cancer medications, it is anticipated that therapies that target them will inhibit cancer from spreading and from reoccurring. Thus, it is crucial in practice to identify the molecules that regulate their function. It has been shown that ceRNAs affect the proliferation and activity of CSCs.

Due to the diversity of miRNA targets, other non-coding RNAs may act as ceRNAs to modulate important gene expression in CSCs. The identification of these ceRNAs could assist the researcher in better understanding tumorigenesis. The discovery of functional ceRNAs in cancer may offer a promising possibility as a treatment approach and diagnostic marker for cancer patients. Cancer therapy should take into account novel signaling pathways or regulatory molecules, including non-coding RNA, RNA editing, epigenetics, and metabolism, as these also promote CSC stemness [[341], [342], [343]]. It is also worthwhile to investigate novel CSC microenvironment targeting strategies. Due to the various signaling pathways improperly expressed in CSCs, combination therapies may have a better effect. A promising approach is to include CSC inhibitors in addition to standard chemotherapeutic agents. These findings and targeting this recently discovered regulatory circuitry may open the path for discovering new cancer therapeutic targets and shed light on understanding novel predictive biomarkers that can be used to guide clinical research.

## Availability of data and materials

No data was used for the research described in the article.

## CRediT authorship contribution statement

**Hamid Aria:** Writing – original draft. **Mahdieh Azizi:** Writing – original draft. **Shima Nazem:** Visualization. **Behnam Mansoori:** Conceptualization. **Farzaneh Darbeheshti:** Writing – review & editing, Conceptualization. **Anoosha Niazmand:** Writing – review & editing. **Abdolreza Daraei:** Supervision, Conceptualization. **Yaser Mansoori:** Supervision.

## Declaration of competing interest

The authors declare that they have no known competing financial interests or personal relationships that could have appeared to influence the work reported in this paper.
